# Green and Low-Cost Natural Lignocellulosic Biomass-Based Carbon Fibers—Processing, Properties, and Applications in Sports Equipment: A Review

**DOI:** 10.3390/polym14132591

**Published:** 2022-06-26

**Authors:** Yueting Wu, Xing Gao, Tat Thang Nguyen, Jie Wu, Minghui Guo, Wenhao Liu, Chunhua Du

**Affiliations:** 1 Graduate School, College of Sports and Human Sciences, Post-Doctoral Mobile Research Station, Harbin Sport University, Harbin 150008, China; wyt1263912258@163.com (Y.W.); wj199703082022@163.com (J.W.); 13936394159@163.com (W.L.); 2 College of Wood Industry and Interior Design, Vietnam National University of Forestry, Xuan Mai, Hanoi 13417, Vietnam; thangnt@vnuf.edu.vn; 3 Key Laboratory of Bio-Based Material Science and Technology (Ministry of Education), College of Material Science and Engineering, Northeast Forestry University, Harbin 150040, China

**Keywords:** lignocellulosic biomass, carbon fiber, spinning-property relations, sports equipment manufacturing

## Abstract

At present, high-performance carbon fibers (CFs) are mainly produced from petroleum-based materials. However, the high costs and environmental problems of the production process prompted the development of new precursors from natural biopolymers. This review focuses on the latest research on the conversion of natural lignocellulosic biomass into precursor fibers and CFs. The influence of the properties, advantages, separation, and extraction of lignin and cellulose (the most abundant natural biopolymers), as well as the spinning process on the final CF performance are detailed. Recent strategies to further improve the quality of such CFs are discussed. The importance and application of CFs in sports equipment manufacturing are briefly summarized. While the large-scale production of CFs from natural lignocellulosic biomass and their applications in sports equipment have not yet been realized, CFs still provide a promising market prospect as green and low-cost materials. Further research is needed to ensure the market entry of lignocellulosic biomass-based CFs.

## 1. Introduction

**Figure 1:**
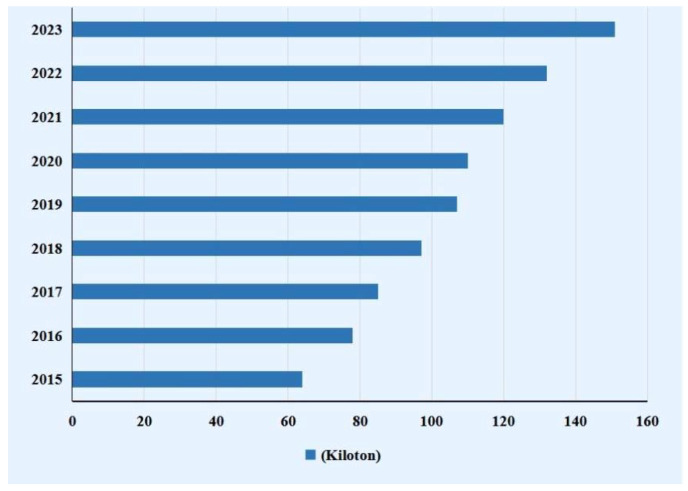
Global demand for CFs.

Carbon fibers (CFs) are special fibers with a carbon content of more than 90%. Their high temperature resistance ranks first among all chemical fibers, and they are known as the “king of new materials”. Most CFs have diameters in the range of 5–10 mm with some notable properties such as high tensile strength, high compressive strength, a high elasticity modulus, low thermal expansion coefficient, and high electrical and thermal conductivity [1,2,3,4]. Due to these superior properties, CFs are widely used in aerospace, automotive, sporting goods, biomedicine, energy generation, military, and other industries, and the global demand for CFs is expected to double every decade [5,6]. Figure 1 shows the global demand for CFs. However, due to the high production cost of CFs (precursor material accounts for about 53% of total production costs), their applications are still limited to manufacturing high-value products in these fields [7].

On an industrial scale, CFs are produced from various precursor materials such as polyacrylonitrile (PAN), pitch, and rayon [8]. PAN fibers are produced through spinning, stabilization, carbonization, and graphitization processes [9]. Industry-recognized spinning techniques include wet-spinning, dry-spinning, and melt-spinning [10,11]. Pitch fibers are manufactured from molten aromatic plastics or petroleum products and also undergo these processes [12,13]. In the 1950s, rayon was one of the first commercially available precursors for CFs. It is a regenerated cellulose fiber that is mixed with a suitable solvent to render it spinnable. After the spinning process, rayon fibers are stabilized and carbonized to form CFs with high tensile strength [14,15,16].

Unlike most polymers, carbon does not melt and cannot be directly extruded into fiber form. Therefore, the precursor material is first processed into precursor fibers (PFs) and then stabilized (the most critical step). The stabilized PFs are heat-treated in an inert environment above 1000 °C to obtain CFs. Currently, high-performance commercial CFs used in structural composite applications are produced almost entirely from PAN. As its main advantage, PAN does not melt; therefore, PFs obtained from PAN can be easily stabilized. Subsequently, the stabilized PFs are carbonized in the range of 1200–1500 °C to yield final CFs with excellent tensile strength (3–7 GPa) and medium-high modulus (200–300 GPa) [17]. However, PAN is expensive (USD33/kg) [5,18,19], and the ethylnitrile groups in PAN can generate toxic by-products such as hydrogen cyanide during the heat treatment process [20]. Therefore, the production of PAN-based CFs is neither an environmentally friendly nor a low-cost process.

**Figure 2:**
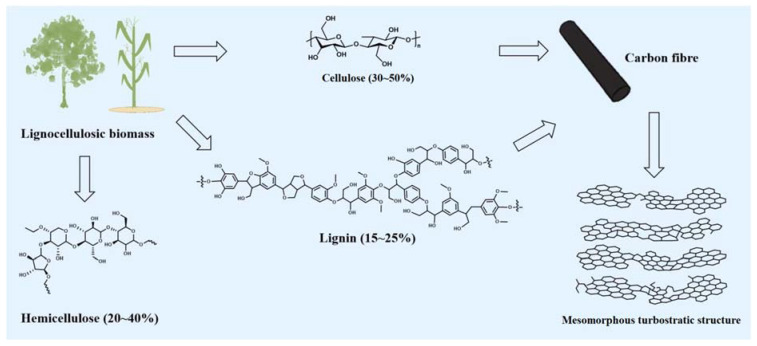
Chemical structures of lignocellulosic biomass and the CFs derived from lignin and cellulose (adapted from Refs. [17,29,30,31,32,34,35]).

In recent years, with increasing environmental awareness, biomass has become more attractive as a precursor to produce CFs [7,9,12,17,21,22,23,24,25]. Biomass is a renewable resource with a wide range of sources which are environmentally friendly and inexpensive. In various industrial applications, environmental pollution, resource shortages, and high costs can be effectively alleviated by using biomass instead of traditional non-renewable petroleum-based materials [26,27,28]. Due to these factors, research on the production of CFs from biomass-based precursors has attracted increasing attention [7,9,12,17,21,22,23,24,25]. As the most abundant biomass resource, lignocellulosic biomass (such as wood, rice husks, corn straw, and forestry waste) is a natural raw material that can be used to produce high value-added products. It is composed of cellulose (30–50% of dry matter weight), hemicellulose (20–40% of dry matter weight), and lignin (15–25% of dry matter weight) [17,29,30,31,32], and also includes a small amount of structural proteins, lipids, and ash [5,17]. Among them, various lignins (60–65%) and celluloses (44.5%) are considered potential precursors of CFs due to their carbon content, low cost, and diversity in chemical structures [33]. Figure 2 shows the chemical structures of lignocellulosic biomass and CFs with a mesomorphous turbostratic structure that were produced from lignin and cellulose [17,29,30,31,32,34,35].

### 1.1. General Schemes and Conditions for Producing CFs

The structure of CFs can be turbostratic, graphitic, or hybrid (containing both turbostratic and graphitic parts) depending on the precursor used to prepare the fibers. For example, CFs derived from PAN have a turbostratic structure with sheets of carbon atoms haphazardly folded or crumpled together. In general, turbostratic CFs possess a high, ultimate tensile strength. Moreover, fibers produced using pitch or rayon as precursors are of lower quality, but their mechanical properties such as modulus or strength can be enhanced by heat treatment. In contrast, CFs derived from mesophase pitch acquire a graphitic structure after heating at temperatures above 2200 °C. These heat-treated CFs possess a high Young’s modulus of elasticity and high thermal conductivity. They exhibit the highest tensile strength (5650 MPa) after heat treatment in the range of 1500–2000 °C (carbonized), and a high modulus of elasticity (531 GPa) after heat treatment at 2500–3000 °C (graphitized).

In general, each carbon filament is formed from a synthetic polymer precursor such as PAN, rayon, or petroleum pitch. The precursors are spun into filament yarns and subsequently arranged via chemical and mechanical processes to enhance the physical properties of the final CFs. After stretching or spinning, the CFs are further processed by heating the polymer filament yarn to remove non-carbon atoms (carbonization). The general scheme for producing CFs involves the heating of the spun PAN filaments to ~300 °C in air. The oxidized PAN is then heated to ~2000 °C in an inert gas atmosphere (graphitization), which changes the molecular bond structure. During the heating process, these chains bond side-to-side (ladder polymers) to form narrow graphene sheets, which eventually merge to form columnar filaments (93–95% carbon content) [36,37].

### 1.2. Precursor Selection

The selection of suitable precursors is the first step in the production of CFs and determines their properties and applications. The suitability of CFs largely depends on the physical and chemical properties of the corresponding precursors [38,39]. To date, a wide variety of precursors such as PAN, rayon, pitch, synthetic polymers, and biomass have been systematically explored. However, not all of these possess the properties required to produce high strength CFs. The greatest challenge for precursors is to meet the three high requirements (high carbon content, high molecular weight, and high purity) of the CF-production process. For example, lignin is an amorphous polymer with poor mechanical properties, and therefore cannot withstand large drafting tensions in the spinning process. As a result, PFs with a larger diameter and lower orientation are obtained. Pitch is an amorphous substance with a low molecular weight and low melting point, and produces CFs with low strength. Rayon fibers and biomass have a carbon content of 44–50%, low degree of polymerization of 250–400, and decreased crystallinity of 30–50%, all of which may lead to low carbon yields, resulting in a low mass of fibers. Therefore, these negative properties of precursors may affect the performance and applications of the final CFs (Table 1).

#### 1.2.1. Polyacrylonitrile

PAN is a high molecular weight polymer polymerized from toxic and carcinogenic acrylonitrile monomer (CH_2_=CH–C≡N), and its specific comonomer content can be as high as 5 wt% [12,53]. In the mid-1950s, the German Bayer company used the copolymer of methyl acrylate and acrylonitrile to prepare PAN fibers which improved the properties of traditional PAN fibers and broadened PAN applications. In the 1940s [9,54], PAN-based PFs were produced by the DuPont company (Wilmington, DE, USA), which were further converted to CFs for the first time by the Government Research Institute of Japan in 1964 [9,54]. This technology was licensed by Japan’s Toray Industries in 1970, which is currently the largest manufacturer of CFs [9,55].

PAN is the most suitable precursor for the production of high-strength CFs due to its molecular properties, high carbon yield, and ability to decompose to form carbon before melting [9]. The molecular structure of PAN contains highly polar propionitrile groups which strongly interact with adjacent groups to form a ladder-like structure, allowing the transformed PFs to maintain their fiber structure during the high-temperature carbonization process [56]. The molecular weight of PAN for textile applications is generally 70–200 kDa [9,57]. PAN used to produce PFs with high crystallinity and high strength should possess high molecular weights (>200 kDa), and comonomers need to be added to achieve a high tensile capacity and better oxygen permeability during spinning and oxidation [9]. Compared to other precursors, such as rayon, pitch, and polyolefins 90% of global CFs production, despite its high cost, depends on PAN due to the excellent comprehensive performance of PAN-derived CFs [9].

While the theoretical strength of a perfect graphite crystal is 100 GPa [1,58], the tensile strength of T1100G CFs currently produced by Toray Industries is only 7 GPa [1,59]. One important reason for the relatively low strength is the existence of structural defects and inhomogeneities in CFs [60]. Thus, the microstructure of PAN-derived PFs, including the diameter, number, size, and distribution of structural defects (surface and pore defects), as well as fiber orientation, morphology, and homogeneity, is critical for the mechanical strength of the final CFs [61,62,63].

Currently, wet spinning is the preferred method for processing PAN-derived PFs [12,53]. The spinning solution is extruded into a void with a high jet stretch ratio, followed by a coagulation process. The high jet stretch ratio acts on the spinning solution in the void, which effectively promotes the arrangement of molecular chains and a reduction in the fiber diameter. After immersion in the coagulation bath, dual diffusion occurs whereby the polymer-rich phase coagulates into the fiber skeleton, while the solvent-rich phase forms voids. After coagulation, the fibers are extracted in a washing bath to promote the alignment and diameter reduction of polymer chains, which has a key impact on the properties of the PFs. 

#### 1.2.2. Pitch

Pitch is a by-product of the coal and oil industries with the advantages of a high carbon content (>80%) and relatively low cost [12]. Pitch fibers are produced by refining, irradiating, pre-oxidizing, carbonizing, and graphitizing petroleum or coal pitch. Compared to PAN, pitch is a low molecular weight material [12]. Therefore, pitch produces CFs with a high modulus of elasticity, almost equal to that of graphite single crystals, which can be higher than that of PAN-derived CFs, but of low strength and can be used for high-modulus applications such as high-quality sporting goods, industrial rollers, and spacecraft [9,64]. Pitch-derived CFs are divided into two categories based on their mechanical properties. One has relatively poor mechanical properties and is produced from isotropic pitch. The other consists of high-performance CFs fabricated from anisotropic mesophase pitch [65] and is used in high temperature structural materials, high thermal conductivity materials, sealing materials, shock-absorbing materials, and aerospace engineering materials.

Pitch is typically heated in an inert atmosphere, whereby a mesophase is established by the condensation of pitch oligomers during this treatment, which is difficult to control [12,66]. However, the mesophase has the advantage that the material can be used in a melt spinning process to produce PFs. During this process, PFs assume a high aromatic layer orientation at the molecular level and can be directly converted to the final CFs. The application of the electrospinning process in the production of pitch-derived PFs requires a high concentration of the pitch solution to achieve sufficient viscosity [8,67]. Tetrahydrofuran has been shown to be a suitable solvent for the preparation of pitch solutions whose application in the electrospinning process can result in PFs with uniform thickness and properties [8,68,69]. Furthermore, the molecular structure of pitch significantly affects its spinnability, and the addition of PAN improves its molecular weight, solubility, and viscosity. Therefore, mixtures of PAN and pitch can be used as precursors for better spinnability [8,68]. 

#### 1.2.3. Rayon

Rayon is a cellulose material produced from natural polymers, such as cotton, wood, and fibrilia, through chemical treatment and machining [70]. However, natural polymers contain numerous hydroxyl functional groups and a high oxygen content in structural units, whose presence lowers the yield of rayon-derived CFs, resulting in the low mass of the fibers [17,71]. In addition, the production of rayon is being phased out due to its high energy consumption and heavy pollution. Rayon can be divided into the following three categories based on its chemical composition: regenerated cellulose fiber, cellulose ester fiber, and regenerated protein fiber. In the early days of CF development, rayon was the standard precursor used to produce CFs due to its ability to decompose before melting. However, rayon-derived PFs have irregular porous cross-sections, resulting in low strength [17,71]. Nevertheless, CFs with a strength of 1.25 GPa and a modulus of 720 GPa can be obtained by graphitizing the stabilized rayon derived PFs at 2800 °C. However, this is an expensive process, which further limits its application [14,16]. 

#### 1.2.4. Synthetic Polymers

Synthetic polymers have been used as precursors of CFs [12,72]. For example, polyethylene, as an inexpensive polymer, can be oxidized and sulfonated by strong acids such as sulfuric acid. Its carbon yield after carbonization (heat treatment up to 1200 °C) is 75%, and the tensile strength and modulus of elasticity of the CFs are 2.5 GPa and 139 GPa, respectively [12,73]. Although high carbon yields can be achieved with such polymers, their resulting CFs have a low tensile strength and poor creep resistance. Other polymers have been reported. Newell and Edie (1996) studied the carbonization of polybenzoxazole and obtained CFs with moderate mechanical properties (tensile strength: 1 GPa, modulus of elasticity: ≤245 GPa) using conventional processes [73,74]. However, such polymers have low carbon yields due to the lack of aromatic groups or easy to form aromatic units and require expensive hot-stretching treatment [66]. 

#### 1.2.5. Biomass

In addition to PAN, pitch, rayon, and synthetic polymers, natural biopolymers or biomass-derived polymers have again become the focus of attention as precursors for CFs. Biomass is an abundant, widely distributed, renewable, and carbon-neutral natural resource, and includes all plants, microorganisms, and animals, as well as their waste products. Biomass has been widely developed in recent years as a precursor for CFs due to its low cost and low pollution during processing [75,76,77,78,79].

### 1.3. Applications of CFs in Sports Equipment

**Figure 3:**
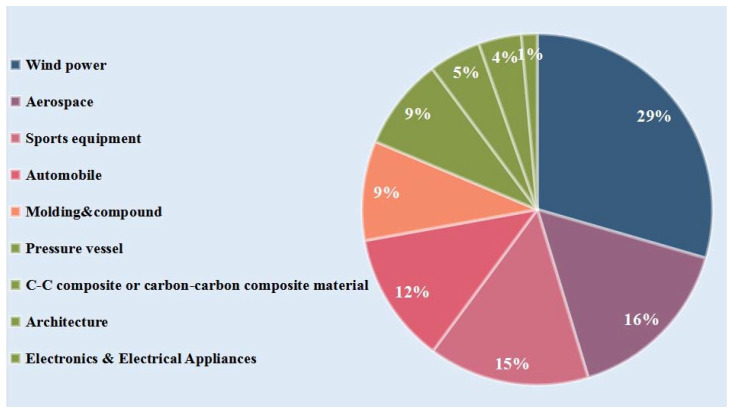
Global demand for CFs in various application fields in 2022 (in kilotons).

Sports equipment is an important medium for the sustainable development of the sports industry [80,81]. Traditional sports equipment is composed of metal, wood, rubber, and leather, and their structural design is relatively simple and lacks functionality, which greatly limits their applications [82,83,84]. With technological development, new materials such as CFs are playing a key role in manufacturing sports equipment. Figure 3 shows the demand for CFs in various application fields [12]. Based on the excellent properties of CFs, such as high specific strength, high specific modulus of elasticity, light weight, corrosion resistance, good damping performance, and designability, the use of CFs has significantly improved the performance of sports equipment. The following section highlights the importance and advantages of CFs in competitive sports. 

#### 1.3.1. Significance of CFs in Competitive Sports

The performance of sports equipment has a significant impact on competition results. Depending on the needs of the different events, sports equipment with good performance characteristics possesses unique advantages in terms of material selection, process design, and ergonomic design, and can significantly contribute to improving the experience and sporting achievements of athletes and ensuring their safety. In recent years, based on developments in carbon fiber materials, sports equipment manufactured from CFs has become a popular choice for competitive sporting applications which enhances the potential of athletes and their confidence in winning [80,81,82,83,84].

**Figure 4:**
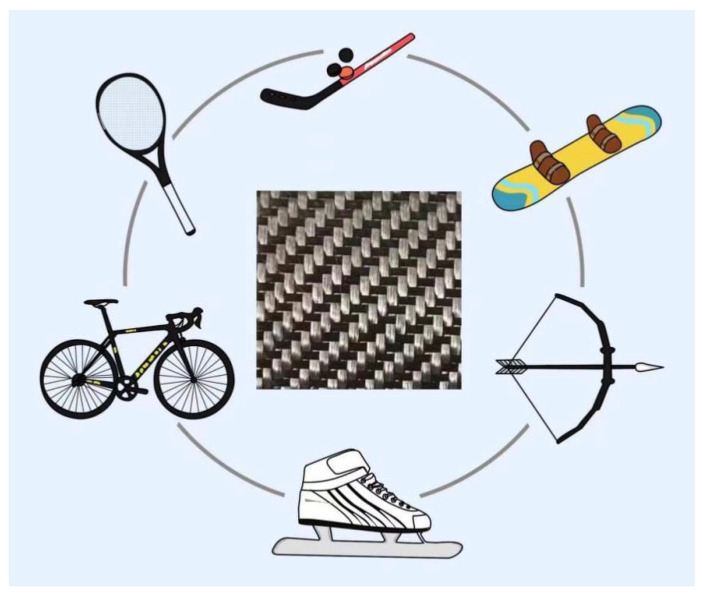
CFC applied in the manufacture of sports equipment.

CFs are used for two important applications in competitive sports, namely direct and indirect applications [85]. Indirect applications are the various material requirements associated with the athletic grounds, referee equipment, information displays, and command communications. Direct applications refer to the various material requirements associated with sports equipment such as sportswear, sports shoes, hats, and sports equipment (training equipment and competition equipment). CFs with their advantage of light weight are suitable materials for sports equipment such as vaulting poles, ball sports equipment (e.g., tennis and badminton rackets), winter sports equipment (e.g., skis, ice hockey sticks, skates), bicycles, archery, and surfboards [85]. Figure 4 shows the carbon fiber cloth (CFC) used in the manufacture of sports equipment, the utilization of which reduces the physical output and improves the competitive performance of athletes.

Traditionally, vaulting poles are made from hickory wood. However, it is difficult for athletes to exploit their kinetic energy due to the high mass, low elasticity, and poor energy storage of wood-based vaulting poles. As an important advancement, vaulting poles made from CFs are lightweight, durable, and show high damping performance and high elasticity, conducive to achieving excellent competition scores. At the Beijing Summer Olympics in 2008, Yelena Isinbayeva, the gold medalist in women’s pole vault, used a vaulting pole with numerous CFs in both head and end that was lightweight and could be bent to 90°. Therefore, her kinetic energy was efficiently converted into gravitational energy and then quickly rebounded, thus assisting her to successfully jump 5.05 m and set a new world record [84]. 

In the mid-1970s, researchers found that, compared with traditional wood-based rackets, rackets made from CFs had advantages of high specific strength, high specific modulus of elasticity, excellent ductility, good shockproof performance, and good slip resistance. The addition of CFs extends the contact time between the ball and the racket, and provides athletes with a comfortable feeling when hitting the ball, assisting them to quickly assess the ball’s initial speed and movement trajectory and then hit it at the most appropriate angle and strength, improving the hit rate [82,84,86].

Winter sports are exciting and often high-risk. Snowboarding is a popular sport and the quality of snowboards directly impacts the safety of the athlete [85]. From a material perspective, there are the three following types of snowboards: wood, metal, and CFs. Traditional wood-based snowboards are inexpensive but easily deformed by moisture, while metal-based snowboards are expensive and less adaptable to winter weather. Compared with wood and metal, snowboards produced from CFs effectively overcome these constraints and have the advantages of lightness, good shock absorption, less deformation, strength, and long service life. Therefore, CFs are the best choice for manufacturing snowboards [83]. CFs also show outstanding advantages for manufacturing surfboards. Because of their unique properties, such as light weight and corrosion resistance, surfboards are less susceptible to seawater corrosion and can achieve faster speeds on the water.

In the 1980s, bicycles produced using CFs were successfully developed in Italy, France, the United Kingdom, and the USA. They were assembled from carbon fiber tubes and aluminum alloy joints, which are attractive and comfortable but are also lighter, stronger, more rigid, and are better at shock absorbing than traditional bicycles. These CF-based bicycles are now used as racing bicycles and are widely accepted in competitive sports [82,83,84].

The first field hockey sticks were fabricated of wood. Due to their low elasticity and large mass, athletes often had difficulty grasping the stick and controlling the direction of the ball. With the development of CF technology, the head of the hockey stick was formed with the hollow forming method, and the center part was filled with a hard material. This design combines the performance advantages of CFs (such as light weight and strength), allowing athletes to use the hockey stick more precisely and to better control the direction of the ball. 

#### 1.3.2. Advantages of CFs Applied in Sports Equipment

Mechanical properties, such as mass, strength, toughness, and elasticity, are important factors to consider when developing sports equipment. Compared to metal and wood, CFs are exceptional in terms of strength, modulus of elasticity, damping performance, and shock absorption properties. Therefore, sports equipment made from CFs can effectively improve the performance of athletes in competitive sports such as tennis, cycling, pole vaulting, skiing, and golf [83].

In comparison to steel and aluminum, CFs and their composites possess higher fatigue strength and maintain more than 65% of this property after numerous tests under stress-fatigue conditions, while steel and aluminum retain about 35% of fatigue strength. Therefore, using CFs is a key factor to prolonging the service life of sports equipment due to their significant difference in fatigue strength [87,88].

Sports equipment produced from traditional materials generally have poor designability, maintenance difficulty, and high maintenance costs due to their limited performance. Therefore, the development of carbon fiber technology offers great possibilities for the design freedom of sports equipment, which is difficult to achieve with wood and metal materials.

The volume and density of the material change to a certain extent when exposed to heat, which corresponds to a thermophysical parameter, i.e., the coefficient of thermal expansion. At room temperature, the coefficients are negative for carbon fiber composites—in temperatures of 200–400 °C, the coefficient of thermal expansion is close to zero; and at temperatures below 1000 °C, the physical dimensions of the carbon fiber composites remain stable. Therefore, the application of CFs can effectively ensure the safety of sports equipment.

The strain capacities in carbon fiber composites are always balanced and similar. Even when parts of the fibers fracture after being subjected to external forces in a radial direction, this will not affect the normal operation of other fibers in the same direction. As a result, when sports equipment manufactured from CFs is subjected to large external forces, it will not completely fracture or affect their overall performance and can enhance the personal safety of athletes [83].

CFs are environmentally friendly, which provides certain guarantees in terms of safety. In addition, some carbon fiber composites can be recovered and recycled, greatly reducing the production cost and is conducive to the further development and promotion of sports equipment. Therefore, the application prospect of CFs in the manufacture of sports equipment have the potential to be very broad in future.

This review describes the latest research on lignocellulosic-based CFs. First, the properties and intrinsic advantages of lignin and cellulose are introduced. Second, several green and effective pretreatment technologies for the separation and extraction of lignin and cellulose from biomass are presented. Third, recent research on the further performance optimization of lignocellulosic-based CFs is summarized. The relationship between different spinning processes and the structure, properties, and applications of the lignocellulosic biomass-based CFs is then discussed. Since there are few studies on CFs derived from lignocellulosic biomass, this review will contribute to this field.

## 2. Lignocellulosic Biomass

Natural lignocellulosic biomass, including wood, crops, grasses, cotton, and straw, is composed of cellulose, hemicellulose, and lignin [17,29,30,31,32]. It represents natural and renewable chemical materials, which may act as precursors to produce high value-added CFs [5,7,89,90]. The various types of lignocellulosic biomass differ in the content, composition, and structure of lignin and cellulose, and can also exhibit different properties. For example, cotton contains up to 98% cellulose, while softwood pulp consists of approximately 90%. In addition, softwoods have a higher lignin content (25–35%) than hardwoods.

Lignocellulosic biomass, in contrast to PAN, contains large amounts of ash and minerals, leading to structural defects in PFs, which are particularly detrimental to the tensile strength of the final product. It is also important to separate and extract lignin and cellulose from lignocellulosic biomass [17]. To maximize their utilization in the production of high quality CFs, a cascade utilization of lignocellulosic biomass is preferred, for which the separation of lignocellulosic biomass into different components is a key step [91]. In recent years, researchers have successfully separated lignin and cellulose in a green and environmentally friendly way, avoiding waste and the use of toxic and harmful substances [92,93], further promoting the properties and sustainable development of lignocellulosic biomass-based CFs. This will be discussed later in this section.

### 2.1. Lignin

**Figure 5:**
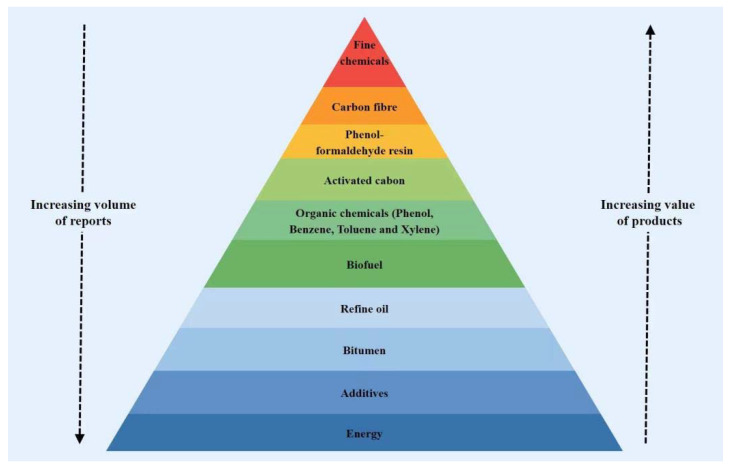
Value-added products manufactured from lignin (adapted from Refs. [5,89]).

Lignin is the second most abundant natural biopolymer after cellulose, which accumulates in millions of tons annually as a by-product of the pulping industry [94,95,96,97]. With its high carbon content and high aromaticity, lignin is an important precursor for the industrial production of functional carbon-based materials, such as activated carbon, CFs, carbon-based catalysts, electrode materials, and carbon black. Among them, CFs are the highest value-added products (Figure 5) [5,98].

#### 2.1.1. Properties of Lignin

The lignin content in wood ranges from 15 wt% to 30 wt% and is lower in hardwoods than in softwoods. It is a cross-linked racemic macromolecule with a molecular structure of oxophenylpropanol or the structural units of its derivatives and is relatively hydrophobic [99]. Lignin is also the only biopolymer that contains a large number of aromatic compounds composed of p-hydroxyphenyl, syringyl, and guaiacyl. The main monomers that form repeating units are p-coumaryl alcohol (H-unit), coniferyl alcohol (G-unit), and sinapyl alcohol (S-unit), formed by several types of C–C (β–1′, β–5′, β–β′, 5–5′) and C–O bonds (β–O–4′, α–O–4′, 4–O–5′) [31,100]. 

#### 2.1.2. Separation and Extraction

The cross-linked structure in lignin must be broken to achieve separation from lignocellulosic biomass, which is carried out through a chemical pulping process. Different pulping methods affect the ability of lignin to produce CFs and the properties of the final CFs [17]. Commercially, the most common and efficient method for obtaining lignin is through kraft pulping, which uses sulfides and alkalis to decompose natural lignin into soluble oligomers [101,102]. However, sulfur is introduced into the separated lignin and can generate defects in the PFs during carbonization. In addition, the process faces environmental problems such as the management of sulfur-containing wastewater. Pulping with organic solvents such as methanol, ethanol, and acetic acid, is a viable option. At present, the mature technology is the Alcell process [17,103] which employs a 50% aqueous ethanol solution to precipitate lignin in the waste liquid without the addition of inorganic substances and sulfur. Due to the higher purity of the separated lignin, the strength of the resultant CFs is enhanced.

The lignin content in the black liquor of the kraft pulping process ranges from 30 wt% to 45 wt%, and can be precipitated and separated from black liquor by acidification followed by several washing cycles [104]. The ash content of the lignin extracted in this process is 0.2 wt% [105]. Ház et al. (2016) separated lignin from black liquor and showed that it possessed great application potential [106]. Klett et al. (2015) reported a process for the separation and extraction of lignin from black liquor using a hot acetic acid solution and successfully obtained “ultrapure” lignin with an ash content of less than 0.1 wt%, which might significantly increase the strength of CFs in future research [107].

Some studies reported on a new method for the separation and extraction of lignin, i.e., microwave-assisted acid pretreatment. For example, Avelino et al. (2018) systematically studied the effect of the acid type on the extraction rate of lignin from coconuts [108]. According to their results, the microwave-assisted acid pretreatment had a higher lignin extraction rate with sulfuric acid (56.6%) than with hydrochloric acid (54.4%) or acetic acid (9.2%). In addition, ionic liquids (ILs) are potential attractive “green” solvents and have been used in an efficient pretreatment system for lignocellulosic biomass [109]. Studies on the utilization of ILs to separate and extract lignin have been reported [110]. It was demonstrated that the pretreatment temperature affects the lignin extraction yield, which improves with increasing temperature. For example, using diisopropyl ethyl ammonium acetate in the extraction of lignin, the yield increased from 24.3% at 80 °C to 71.2% at 120 °C [111]. 

#### 2.1.3. Lignin-Based CFs

Lignin has always been regarded as a promising low-cost precursor for CFs and was first used in their production in as early as the late 1960s [12,112]. Lignin-derived precursor fibers maintain the original filamentous morphology during the pre-oxidation process due to the aromatic hydrocarbon structures. In addition, due to the interactions of π–π bonds between the aromatic hydrocarbons, the resultant PFs exhibit not only better thermal stability but can withstand higher heating rates during the pre-oxidation process, thus increasing the yield of CFs and reducing energy consumption. Lignin is a thermosetting compound; therefore, it decomposes rather than melts during the heating process. However, the products of its modification [113] or decomposition, such as lignin-based pitch [114], can be placed in a melt-spinning process. In addition, the spinning solutions of lignin in organic solvents can be extruded in the process [115]. For example, Sano et al. used purified organsolv lignin without sulfur or other components to prepare CFs via a melt-spinning process. The cost of production and the tensile strength (0.3 GPa vs. 3.5 GPa) are much lower than those of conventional PAN-based CFs [44,116].

The major challenge in the development of lignin-based PFs and CFs is the spinnability of lignin, which is affected by the atomic and molecular bonds, conformation and configuration of molecular chains, degree of order and disorder of molecular chains, molecular weight and its distribution, and other factors which influence the thermal behavior of lignin in the melt-spinning process. For example, Zhou et al. [89] connected lignin and cellulose acetate (CA) by covalent bonds via cross-linking agents and detailed the thermo-stabilization of the mixed spinning solutions. With the introduction of the covalent-bond connection, the PFs exhibited excellent thermal stability. Hardwood lignin is a more suitable precursor than softwood lignin which has no softening temperature point and therefore cannot be used for melt-spinning. In addition, the solubility and molecular weight of lignin in a solvent, the rheological properties of the lignin solution, solvent type, and solvent volatility are all key factors in wet-spinning and electrospinning processes [5]. 

### 2.2. Cellulose

Cellulose is an abundant biopolymer and present in the structural components of plant cell walls. Wood contains 40–50% cellulose [94,117]. In 1838, Payen identified and named cellulose [118] and cellulose has become an ideal precursor for the production of CFs.

#### 2.2.1. Properties of Cellulose

Cellulose is a linear polymer composed of several hundred to tens of thousands β–(1→4) glycosidic bond-linked glucose units, which form anhydroglucose units [119]. During biosynthesis, van der Waals forces and intermolecular hydrogen bonds promote the parallel alignment of cellulose chains, forming basic fibers at a nanoscale, which further organize into larger and stable fibers. In addition, the cellulose chains arrange in highly ordered crystalline regions as well as in amorphous or disordered regions. Therefore, cellulose is insoluble in water and in common organic solvents due to the coexistence of ordered and disordered regions. It can be divided into cellulose type I and cellulose type II. Natural cellulose is only cellulose type I, whereas cellulose type II is formed after the treatment of natural cellulose with concentrated alkali solutions or after regeneration from cellulose solutions. For example, rayon fibers contain cellulose type II. In general, cellulose type II has a more stable crystal structure and can be produced by redeposition and mercerization. Cellulose type I consists of two polycrystalline structures, I_α_ (triclinic structure) and I_β_ (monoclinic structure). Cellulose I_α_ crystallizes into a triclinic lattice with one polymer chain in the unit cell. Cellulose I_β_ crystallizes in a monoclinic lattice and contains two polymer chains per unit cell. Dimorphism occurs when two-phase cellulose type I coexists in the same natural cellulose sample or even the same microfibril, which minimizes the loss of its overall crystallinity [120,121,122].

#### 2.2.2. Separation and Extraction

Supercritical fluid (SC) is a low-cost reagent with a low critical temperature and pressure, low viscosity and is non-toxic, recyclable, and environmentally friendly [123]. Supercritical carbon dioxide (SC-CO_2_) technology has been widely used in the pretreatment of lignocellulosic biomass to produce cellulose. During the pretreatment process, CO_2_ molecules penetrate the micropores of lignocellulosic biomass, resulting in physical changes and destroying its structure through the subsequent rapid release of CO_2_ [124]. The utilization of a mixed solvent consisting of SC-CO_2_, water, and ethanol has a stronger pretreatment effect than SC-CO_2_ alone. Lü et al. (2013) pretreated corn straw with SC-CO_2_/water, SC-CO_2_/ethanol, and SC-CO_2_/water/ethanol [123] and found that the compact matrix structure of corn straw could be most effectively destroyed using SC-CO_2_/water/ethanol, by highly exposing the cellulose. A cellulose content of up to 74% could be achieved after pretreatment.

Ultrasonic irradiation is a novel pretreatment technology of lignocellulosic biomass, which can reduce the pretreatment time and increase energy efficiency. Ultrasound radiation degrades the cell walls of lignocellulosic biomass and breaks the chemical bonds that connect lignin, hemicellulose, and cellulose [125]. A higher content of cellulose can be obtained by ultrasonic-assisted alkali pretreatment technology, which destroys the structure of lignocellulosic biomass and promotes the diffusion of alkali into the interior of the material, thus promoting the separation of cellulose. Manasa et al. (2018) used ultrasonic-assisted sodium hydroxide to pretreat fibrilia and obtained 68% cellulose [126]. The results showed that the separation effect of ultrasonic-assisted alkali pretreatment was better than that of alkali pretreatment alone.

#### 2.2.3. Cellulose-Based CFs

Cellulose has an important property required for electrospinning to produce CFs, which is its good flexibility. However, it is difficult to obtain CFs directly from natural cellulose through carbonization due to its low heat resistance. Researchers have obtained two kinds of nanocellulose (NC) from cellulose with chemical or mechanical treatment, cellulose nanofibers (CNF) and cellulose nanocrystals (CNC). NC is an ideal precursor to produce CFs with the advantages of a unique structure, excellent mechanical properties, and a low thermal expansion coefficient. It has been reported that NC extracted from wood pulp by acid hydrolysis was mixed with PAN to form PAN/NC blends, acting as a precursor to produce CFs [127]. In addition, the University of Queensland in Australia deconstructed nettles to extract NC, which was then mixed with PAN to produce CFs [9,15]. These methods can effectively improve the grain size and tensile properties of the final CFs. However, the quality of these CFs is still low. 

### 2.3. Performance Optimization of Lignocellulosic Biomass-Based CFs

Research on the production of CFs from lignocellulosic biomass is still in its preliminary stages. Some strategies have been developed to enhance the performance of these CFs, such as blending with PAN/acrylonitrile (AN) [128,129], carbon nanotubes (CNTs) [130], synthetic thermoplastic polymers [131,132], lignin, and cellulose [48,133].

#### 2.3.1. Blending with AN/PAN/CNTs

In some studies, AN, PAN, or CNTs were blended with lignin or cellulose to improve the performance of the final CFs. For example, Maradur et al. (2012) used hardwood lignin and AN for a two-step radical polymerization to form a lignin–AN copolymer, which was then dissolved in dimethyl sulfoxide to prepare PFs via a wet-spinning process [134]. In addition, Ramasubramanian (2013) blended butyrated softwood kraft lignin (SKL) and organic solvent lignin with PAN to form copolymers which were converted into PFs in a wet-spinning process [49]. However, the properties of these final CFs were not reported. Xia et al. (2016) used a lignosulfonate-AN copolymer as a precursor to prepare PFs with a dense structure without visible voids and defects via a wet-spinning process. However, the average strength of the final CFs was only 540 MPa [135]. Wang et al. (2016) grafted lignin onto CNTs (CNTs-g-L) to enhance the mechanical properties of lignin-based CFs. However, numerous voids were generated in fibers obtained from a mixture of lignin and CNTs-g/L during the carbonization process due to the fracture of the chemical bonds between CNTs and lignin. However, at a CNTs-g-L content of 0.5%, the tensile strength of CFs increased from 171.2 MPa to 289.3 MPa [5,130]. Researchers have been able to control the generation of voids by optimizing the stabilization and carbonization processes [5].

#### 2.3.2. Blending with Other Synthetic Polymers

Fluidity may be improved by adding synthetic thermoplastic polymers such as polyethylene oxide, polyethylene glycol terephthalate, polypropylene, and polylactic acid to the lignin precursors [131,132]. For example, Kubo and Kadia (2005) converted hardwood lignin into CFs using such additives. However, the strength and modulus of elasticity of the final CFs were low (703 MPa and 94 GPa, respectively) due to the presence of voids [136]. Lin et al. (2012) obtained softwood lignin with good fluidity by dissolving wood chips in polyethylene glycol and sulfuric acid. However, the tensile strength of the CFs was low (460 MPa) due to the porous and relaxation structure present within the PFs and the difficulty to stabilize them. Even though chemical treatment could increase the strength to 700 MPa, the harsh treatment conditions could not sustain the environmental advantages of using lignocellulosic biomass as a precursor [137]. Researchers have also tried to add other solid components such as organic clay. However, the CFs produced with a strength of 500 MPa were not appropriate for structural applications [138]. Recent studies have reported the utilization of ILs to produce cellulose based PFs. Imidazolium-based ions combined with various cations and anions were used as the solvent, blending cellulose (cotton and viscose) with synthetic polymers (polyester, polyamide, or PAN). For example, Ingildeev et al. (2012) developed a method that used ILs for producing cellulose/PAN fibers. The two components were blended to prepare a spinning solution, finally obtaining PFs via a wet-spinning process. The results showed that its carbon yield was higher than that of pure cellulose fibers [139]. 

#### 2.3.3. Lignin–Lignin Blends

Several research institutes have proposed improvements in the processing and performance of single lignin-based CFs by blending hardwood lignin with softwood or plant lignin. For example, Oak Ridge National Laboratory (ORNL) focused on blends of hardwood and softwood lignin and reported a lignin-derived CF with a tensile strength of about 0.5 GPa and Young’s modulus of 29 GPa [12,140]. Furthermore, the addition of switchgrass lignin to boxwood lignin could significantly enhance the spinnability of boxwood lignin by improving its thermal stability, avoiding the fusion of PFs during the fast stabilization process. The mechanical properties of the final CFs were enhanced, exhibiting, with diameters of 16–32 μm, tensile strengths of 230–750 MPa and a Young’s modulus of 30.4–41.8 GPa [5,48]. These results demonstrated a strategy for procuring pure lignin-derived CFs, which can significantly reduce the required production time and improve mechanical properties.

#### 2.3.4. Lignin–Cellulose Blends

To overcome the shortcomings of cellulose and lignin, researchers blended the two materials to form composite fibers in order to improve the properties of single lignin- or cellulose-based CFs. Ma et al. (2015) co-dissolved cellulose and lignin in an IL solvent (1,5-diazabicyclo[4.3.0]non-5-enium acetate) to form a homogeneous solution and then produced high-performance PFs via a dry-jet wet-spinning process [141]. Olsson et al. (2017) and Bengtsson et al. (2019) reported that they successfully converted cellulose–lignin composite fibers into CFs with tensile strengths of 780 MPa and 1070 MPa, respectively [142,143]. Byrne et al. (2014) blended cellulose with lignin, obtaining CFs with a hollow structure and high surface area after the carbonization process, because the degradation rate of cellulose chains was slower with the introduction of lignin, which provides a prospect for applications in batteries as catalyst carrier or in membranes [144].

**Figure 6:**
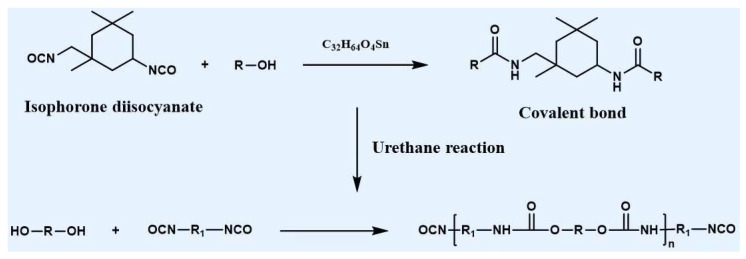
Reaction mechanism of connecting lignin and cellulose via covalent bonds (adapted from Ref. [80]).

Currently, pure lignocellulosic biomass-based CFs have been successfully synthesized from lignin–cellulose blends. However, the performance of these CFs is still not satisfactory, which is related to their morphological collapse due to differences in the physicochemical properties (such as solubility, plasticity, and oxygen content) of lignin and cellulose [89]. Lignin is characterized by hydrophobicity, poor plasticity, and a low oxygen content, while cellulose possesses hydrophilicity, excellent flexibility, and a high oxygen content [145]. The physical blending of lignin and cellulose can lead to the presence of weak intermolecular interactions, resulting in notable phase separation during the thermal stabilization process [146]. In addition, in contrast to cellulose, lignin has a lower thermal weight loss rate during carbonization due to its lower oxygen content. The different thermal degradation rates of lignin and cellulose and the phase separation between these materials lead to the formation of many defects on the surface of CFs [147]. Inspired by natural wood, Zhou et al. proposed the use of chemical modifiers such as isophorone diisocyanate to form covalent bonds between lignin and cellulose, and successfully prepared pure lignocellulosic biomass-based PFs with a high molecular weight, uniform molecular weight distribution, excellent thermal stability, and good spinnability [89]. Figure 6 describes the mechanism of this modification reaction. The covalent bonds effectively maintained the morphology of CFs, which significantly contributed to enhancing their strength. Until recently, the mechanical properties of these CFs were still inferior to those of PAN-based CFs, but such studies constitute a meaningful and promising start to the development of pure lignocellulosic biomass-derived CFs.

#### 2.3.5. Blending with Other Biomasses

In recent years, biopolymer blending has gained increasing popularity as a convenient and inexpensive approach to produce CFs with good mechanical properties. Research has demonstrated that various biomass-containing compositions can be used as potential renewable precursors to produce CFs (Table 2). For example, polylactic acid (PLA) is a biopolymer derived from corn starch and sugarcane biomass and blending lignin with PLA provides a low cost alternative precursor. Thunga et al. (2014) chemically modified softwood kraft lignin via a butylation reaction and blended it with PLA, which producing CFs in a melt-spinning process [45]. The results showed that chemical modification enhanced the miscibility of lignin with PLA, resulting in final CFs with moderate mechanical properties (tensile strength of 10 MPa and Young’s modulus of 20 MPa). Zhu et al. [148] obtained CFs with tensile strengths of 258.6–159.2 MPa and a Young’s modulus of elasticity of 1.7–11.6 GPa using lignin/PLA blends via a melt-spinning process. The introduction of PLA improved the spinnability of lignin/PLA, resulting in the production of continuous lignin/PLA fibers.

Recently, Peng et al. [149] fabricated composite filaments composed of lignin and cellulose nanofibres (CNFs) via a microfluidic spinning process and in situ interfacial complexation techniques. The hierarchical assembly of well-ordered lignin/CNFs crosslinked using biomass chitosan via ionic bonds was stabilized and carbonized, and resulted in the formation of biomass-based CFs with fine graphitic microcrystals. When the lignin content was 75 wt%, the CF tensile strength could reach 1648 MPa, which exceeded most values reported in the literature. Yang et al. (2018) developed self-standing nitrogen-doped CF networks from plant protein–lignin precursors [150]. The challenge encountered during their preparation was to maintain the fibrous structure during the carbonization process. The results showed that at protein-to-lignin ratios of 50:50 to 20:80, electro spun fibers could retain their fibrous structure after carbonization. This research provided the scope to add value to plant proteins and lignin as by-products of agricultural processing. However, low carbon yields are a major limitation to the use of cellulose as a carbon fiber precursor. Zahra et al. [151] used biomass chitosan as a natural charring agent and obtained cellulose–chitosan composite fibers in a dry-jet wet spinning process, which significantly improved the carbon yield of cellulose-derived CFs.

The possibility of low-value lignocellulosic streams (e.g., municipal waste, forest brush, rice straw) and liquid wood precursors has been investigated. They represent an interesting class of precursors, capable of providing a low-cost method for producing CFs. For example, Ma and Zhao (2011) mixed powdered wood in phenol and added phosphoric acid at 160 °C for 2.5 h [152]. The liquid was then polymerized by the addition of hexamethylenetetramine (5 wt%) and heated to induce crosslinking. The solution was then spun, and the fibers stabilized. The results showed that the final CFs had a tensile strength of 1.7 GPa and Young’s modulus of elasticity of 176 GPa.

## 3. Relationship between Spinning and Properties of Lignocellulosic Biomass-Based CFs

**Figure 7:**
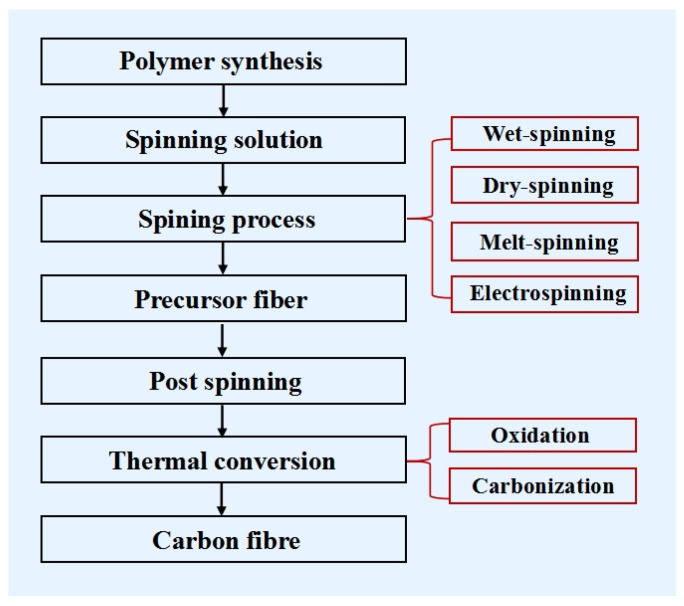
Flow chart of the CF production process.

Figure 7 shows the four steps in the production of CFs: polymer production, spinning, post-spinning, and thermal conversion (oxidation and carbonization) [9]. High-strength CFs used in structural applications must exist in an uninterrupted form to meet the needs of mechanical reinforcement. Therefore, an appropriate spinning process must be selected to produce a continuous form of PFs, which can be finally converted into CFs [8,17,27]. The preferred general technical scheme and condition to produce CFs from lignin involves the preparation of a suitable lignin precursor, which is spun into fibers in an inert atmosphere. The lignin fibers are then subjected to oxidative thermostabilization and carbonization. The integrity of the lignin fiber during oxidative thermostabilization depends on its cross-linking ability. The process is complex and requires careful control of the lignin content, spinning conditions, treatment temperatures, and ramping profiles to obtain CFs with excellent strengths. The preparation of CFs from lignin offers advantages over other precursors such as PAN, as it is inexpensive, renewable, and is already substantially oxidized. Therefore, it can be oxidatively thermostabilized at a significantly higher rate than PAN. Currently, the spinning processes used in industry is divided into the following four types: wet-spinning, dry-spinning, melt-spinning, and electrospinning. Based on recent research, the relationship between the four spinning processes and the properties of the resulting lignocellulosic biomass-based CFs are outlined.

### 3.1. Wet-Spinning

Wet-spinning is the traditional spinning process most used in industry, which is also the preferred method for manufacturing high-strength CFs [12]. As one of the production advantages of this process, PFs are not required to be thermally stable since the residual reactivity can be effectively used for their fast cross-linking and thermal oxidation stabilization, thus allowing for their successful carbonization [17]. However, high production costs due to the need for additional solvent treatment is a major problem [12]. Other spinning processes, such as dry-spinning, melt-spinning, and electrospinning, usually produce CFs with voids and surface defects.

Since cellulose can be dissolved in solvents, their PFs can be produced via wet-spinning. Lignin (such as SKL) recovered from the pulping process does not possess a long and linear molecular structure and therefore have an insufficient tensile viscosity, but it can be converted into PFs by wet-spinning [17]. Some studies blended lignin with synthetic polymers to produce PFs via wet-spinning [153,154,155]. For example, Dong et al. (2015) used lignin sulfonate/PAN blends as candidate precursors in the production of low-cost CFs via a wet-spinning process [153]. However, the microporous structures in the PFs affected the properties of CFs. Ogale et al. (2016) developed a simple and low-cost method for controlling the coagulant composition to balance the counter-diffusion rate of lignin during the wet-spinning process, which eliminated microvoids in fibers and successfully produced void-free lignin/PAN-based CFs [17]. Table 3 shows the different spinning processes for producing various lignocellulosic biomass-based CFs. As shown in this table, Husman (2014) added PAN of a higher molecular weight to lignin (25 wt%) to reduce the porosity of the fibers and used wet-spinning to manufacture PFs, ultimately obtaining CFs with an average tensile strength of 2.25 GPA and a tensile modulus of 217 GPa [155]. These are the highest values for CFs produced using lignin–PAN blends.

### 3.2. Dry-Spinning

In this process, the polymer fiber is precipitated after being pulled into the bath solution and forms a one-dimensional structure by solvent washing and curing after evaporation. Dry-spinning is a technique wherein the polymer solution is sprayed into the air, rather than treated by the coagulating bath, and subsequently is solidified by airflow [17]. Otani et al. (1969), using water as a solvent and adding sodium hydroxide to dissolve the lignin, produced PFs via dry spinning and obtained CFs with a tensile strength of 800 MPa. However, due to residual sodium hydroxide during the carbonization process, defects were present in the final CFs [112,156]. Ogale et al. (2016) employed acetone as a solvent and converted partially acetylated SKL into CFs by dry-spinning. The results showed that the PFs formed crenulated surface patterns due to the out-diffusion of acetone during solidification. In addition, they stabilized and carbonized these lignin-based PFs under tension, resulting again in CFs with a crenulated surface. Due to the enhanced carbon layer plane orientation in the resulting fibers, the tensile modulus and strength values of such CFs can reach 52 ± 2 GPa and 1.06 ± 0.07 GPa, respectively (Table 3), which are the highest values for lignin-based CFs reported in the literature [17,46].

### 3.3. Melt-Spinning

Melt-spinning is an inexpensive, high-throughput, and environmentally friendly process of producing PFs without using solvents. During this spinning process, the molten polymer is extruded through a spinneret with small holes (100–500 μm) and subsequently stretched into fine fibers, which are air cooled and then cured. However, internal voids and surface defects are present in the PFs produced via melt-spinning [17]. Therefore, this process must be optimized to produce high-quality CFs in future.

Since the 1990s, the production of various CFs by melt-spinning from the products of modification or thermolysis of lignin has attracted considerable attention. Sudo and Shimizu (1992) first used methanol to extract lignin from steam-exploded birchwood and modified it into a fusible material using hydrogenolysis, resulting in CFs with a tensile strength of 660 MPa and a tensile modulus of 40.7 ± 6.3 GPa. However, this is not an environmentally friendly nor economical production strategy due to the addition of chemical treatments in the process [43]. Qin and Kadla (2012) fabricated CFs from the products of thermolysis of lignin by melt-spinning. However, these CFs had relatively low tensile strengths (370 MPa) due to the presence of large voids in the PFs [157]. Baker and Rials (2013) proposed an effective method to accelerate the thermal oxidation stabilization step which improved the mechanical properties of the CFs (Table 3). Organically purified hardwood kraft lignin was first heat-treated, and CFs with a tensile strength of 1.07 GPa were obtained by melt-spinning, which was the highest strength of CFs obtained from lignin-derived precursors via this method [76]. Compared with kraft lignin and lignosulfonate, the organic solvent for lignin does not require the addition of metals or sulfur during the separation process, resulting in a more easily spinnable material due to the advantages of high purity, relatively low glass-transition temperature, and stable melt viscosity.

### 3.4. Electrospinning

Electrospinning is different from mechanically driven spinning processes and uses the interaction between a charged polymer and an external electric field to exert high pressure on the polymer solution for spraying in the form of fibers, which is a convenient and easily applicable spinning process [9]. Compared to conventional spinning techniques, the PFs produced by electrospinning are thin (~0.3 μm), short, and are collected as fiber mats rather than as continuous fibers [158]. These fiber mats are used in a wide range of applications and have certain advantages, such as a large specific surface area, high conductivity, and flexibility [159]. However, only a few reviews have been published on CFs produced from lignocellulosic biomass with electrospinning. Nar et al. (2016) discovered a new C-lignin, poly(caffeoyl alcohol) (PCFA) in seeds of the vanilla orchid. They subsequently produced PCFA-derived CFs using an electrospinning process without additional chemical modification or blending with polymers. These CFs were smaller in diameter (10 μm) and structured more orderly than the kraft lignin-derived CFs (50 μm) [5,160].

## 4. Conclusions

Lignocellulosic biomass has the advantages of natural abundance, renewability, low cost, and low environmental impact. Therefore, the production of CFs from lignocellulosic biomass (lignin and cellulose) and the further optimization of their properties offers an alternative to limited petroleum-based resources and provides opportunities for their subsequent expansion to a commercial scale. However, the following inherent properties of lignin and cellulose present major obstacles to the production of high-performance CFs: (1) CFs produced directly from lignocellulosic biomass have a high ash content which limits their application. Thus, the cascade utilization of lignin and cellulose is a potential way to produce high-performance lignocellulose-based CFs. (2) PFs converted from lignocellulosic biomass cannot presently meet the requirements for high strength, and blending such precursors with AN, PAN, and other polymers may be effective to obtain CFs with enhanced properties. However, environmental issues similar to those of petroleum-based materials will still exist. Inspired by nature, blending lignin with other biomasses via a chemical modification technology has been proven to be an effective strategy for preparing green, low-cost pure lignocellulosic biomass CFs. (3) The properties of CFs are sensitive to the spinning process and the PFs used. However, information on the effects of different spinning processes on PFs as well as on the final CFs is still limited. In future, (i) the diversity of lignocellulosic biomass materials should be further investigated; (ii) further modification of the physicochemical properties of biomass precursors and optimization of the processing technology for continuous fibers will be required to overcome limitations in order to produce higher quality lignocellulosic biomass-based CFs for commercial applications; and (iii) in-depth explorations of the key attributes of natural biomass to produce CFs with sufficient mechanical properties are required. Although the lignocellulosic biomass-derived CFs are currently not comparable to CFs from petroleum-based materials in terms of mechanical properties, they provide a potential new market as environmentally friendly materials with low production costs.

## Figures and Tables

**Table 1:** Comparison of the different CF precursors and the properties of the final CFs.

Carbon Fiber Precursor	Carbon Content (%)	Degree of Crystallinity (%)	Tensile Strength of CFs (GPa)	Modulus of CFs (GPa)	Refs
PAN	67.81	35–50	3–7	100–500	[40,41]
Pitch	81.7	-	1–3	200–800	[40,41]
Rayon	62.1	30–40	0.5–1.2	40–100	[42]
Polyethylene (PE)	85.7	55–65	2.5	139	[12]
Polybenzoxazole (PBO)	70.6	55–58	1	245	[12]
Various types of lignin	60–65	-	0.15–0.8	N/A	[4]
Steam-exploded hardwood lignin	-	-	0.66 ± 0.23	40.7 ± 6.3	[43]
Softwood kraft lignin	55.1	-	1.06 ± 0.07	52 ± 2	[17]
Birch wood lignin	63.7	-	0.66	40.7 ± 6.3	[43]
Organosolv hardwood lignin	64.3	-	0.355 ± 0.053	39.1 ± 13.3	[44]
Hardwood kraft lignin	58.5	-	0.52 ± 0.182	28.6 ± 3.2	[45]
Acetylated softwood kraft lignin	61.3–62.8	-	1.06 ± 0.07	52 ± 2	[46]
Softwood/hardwood kraft lignin	63.8	-	0.233–0.377	25–33	[47]
Switchgrass/boxwood lignin	60.3	-	0.23–0.75	30.4–41.8	[5,48]
Lignin (25%)/PAN blend	65.1	-	2.25	217	[49]
Lignin (30%)/PAN blend	64.5	-	1.72 ± 0.2	230 ± 7	[50]
Lignosulfonate-AN copolymer	40.4–48.5 (500 °C)	-	0.54	-	[51]
Hardwood kraft lignin/PEO	57.3–59.7	-	0.458 ± 0.097	59 ± 8	[52]

**Table 2:** Production and properties of CFs from composites containing various biomasses.

Carbon Fiber Precursor	Spinning Process	Mechanical Property	Advantage of Characteristic	Refs
Modified softwood kraft lignin/PLA	Melt-spinning	Tensile strength of 20 MPa Modulus of elasticity 10 MPa	Enhanced the miscibility	[45]
Lignin/PLA	Melt-spinning	Tensile strength of 258.6–159.2 MPa Modulus of elasticity 1.7–11.6 GPa	Increased the spinnability	[148]
Lignin/CNF–chitosan	Microfluidic spinning	Tensile strength of 1648 MPa	high orientation degree and compact microstructure of the filament	[149]
Plant protein–lignin	Electrospinning	-	well-engineered structural characteristics	[150]
Cellulose–chitosan	Dry-jet wet spinning	Modulus of 22.2 ± 1.3 GPa	Improved the carbon yield and structural properties	[151]

**Table 3:** Different spinning processes for producing lignocellulosic biomass-based CFs.

Carbon Fiber Precursor	Spinning Process	Set-Up	Property	Advantage	Refs
lignin (25 wt%)/PAN	Wet-spinning	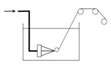	Tensile strength of 2.25 GPa Modulus of 217 GPa	Reduced fiber porosity	[155]
Acetylated softwood kraft lignin	Dry-spinning	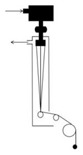	Tensile strength of 1.06 ± 0.07 GPa Modulus of 52 ± 2 GPa	Enhanced carbon layer plane orientation of the fibers	[17,46]
Modified hardwood kraft lignin	Melt-spinning	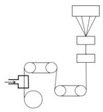	Tensile strength of 1.07 GPa	Improved mechanical properties	[76]
Lignin–cellulose acetate blends	Electrospinning	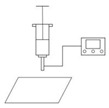	Tensile strength of 49 ± 4 MPa Modulus of 3.0 ± 0.5 GPa	A large molecular weight, uniform molecular weight distribution, excellent thermal stability, and good spinnability of the PFs	[89]

## Data Availability

Not applicable.
